# Vimentin and Notch as biomarkers for breast cancer progression

**DOI:** 10.3892/ol.2014.1781

**Published:** 2014-01-03

**Authors:** GLORIA M. CALAF, ADAYABALAM S. BALAJEE, MARIA T. MONTALVO-VILLAGRA, MARIANA LEON, NAVARRETE M. DANIELA, RAÚL GONZÁLEZ ALVAREZ, DEBASISH ROY, GOPESHWAR NARAYAN, JORGE ABARCA-QUINONES

**Affiliations:** 1 Institute for Advanced Research, Tarapacá University, Arica 8097877, Chile; 2 Center for Radiological Research, Columbia University Medical Center, New York, NY 10032, USA; 3 Departament of Health Science, Faculty of Science, Tarapacá University, Arica 8097877, Chile; 4 Department of Pathology, Dr. Gustavo Fricke Hospital of Viña del Mar, Valparaiso 2581907, Chile; 5 Department of Natural Sciences, Hostos College of the City University of New York, Bronx, NY 10451, USA; 6 Department of Human Molecular Genetics, Banaras Hindu University, Varanasi 221005, India; 7 IREC, School of Medicine, IREC, St. Luc Hospital, University of Louvain, Brussels 1200, Belgium

**Keywords:** biomarkers, estrogen, radiation, breast cells, vimentin, Notch

## Abstract

Breast cancer, the most common spontaneous malignancy diagnosed in women, is a classical model of hormone dependency as it is associated with prolonged exposure to female hormones. Different cytoplasmic proteins are important in the transformation of a normal cell to an invasive tumor cell, and these include vimentin and Notch. To investigate the importance of these two genes and proteins in breast carcinogenesis, we used an *in vitro* breast cancer model system, in which an immortalized human breast epithelial cell line, MCF-10F, was malignantly transformed by exposure to low doses of high linear energy transfer α particle (150 keV/μm) radiation and subsequent growth in the presence or absence of 17β-estradiol. This model consisted of human breast epithelial cells in different stages of transformation: i) a parental cell line (MCF-10F), ii) an Estrogen cell line (MCF-l0F continuously grown with estradiol at 10^−8^), iii) a malignant and non-tumorigenic cell line (Alpha3), iv) a malignant and tumorigenic cell line (Alpha5) and v) a Tumor2 cell line derived from a xenograft of the Alpha5 cell line injected into nude mice. Vimentin and Notch showed greater expression in the Alpha5 and Tumor2 cell lines compared with that in the non-tumorigenic cell lines, MCF-10F, Estrogen and Alpha3. In the present study, positive staining for vimentin was found in 21% of cases. Vimentin and Notch protein expression was negative in noninvasive ductal carcinoma biopsies from breast cancer patients. However, positive cell expression was observed in invasive ductal carcinoma biopsies. These biomarkers can be considered important indicators of breast cancer progression and can be added to the diagnostic panel when overall survival is a primary end-point.

## Introduction

Breast cancer, the most common spontaneous malignancy diagnosed in women, is a classical model of hormone dependency. There is evidence that breast cancer risk is associated with prolonged exposure to female hormones, as the onset of menarche, late menopause and hormone replacement therapy are associated with greater cancer incidence (1). The progression of breast cancer follows a complex multi-step process that depends on various exogenous (diet and breast irradiation) and endogenous (age, hormonal imbalances, proliferative lesions and family history of breast cancer) factors (2–4). Breast cancer is a complex disease in which numerous genetic aberrations occur. Cellular and molecular changes that occur during the development of cancer can be mediated by a range of endogenous and environmental stimuli. On the basis of the currently accepted view of breast cancer as a multi-step process, it is possible that specific abnormalities may be an essential part of the transformation of a normal cell to an invasive tumor cell.

Different cytoplasmic proteins are key in the transformation of a normal cell to an invasive tumor cell and among these, vimentin is particularly important. It is one of the cytoplasmic intermediate filament proteins, which are the major components of the cytoskeleton normally found in embryonic or mesenchymal stem cells (5,6). However, vimentin is frequently expressed in neoplastic cells with metastatic properties, including breast cancer cells (7,8). It is a 57-kDa intermediate filament protein, which forms a part of the cytoskeleton. Expression of vimentin and cytokeratins has also been described in breast carcinomas. Hendrix *et al* (9) demonstrated that the co-expression of vimentin and keratin intermediate filaments in human breast cancer cells results in phenotypic inter-conversion and increased invasive behavior.

Another important gene, *Notch*, is also pivotal in this context. This gene is expressed in a variety of tissues, indicating that it is involved in multiple signaling pathways (10–14). It is either overexpressed or rearranged in human tumors, such as is the case with the 280- to 330-kDa Notch protein (14). The *LIN-12/Notch* family of transmembrane receptors is believed to be central to development by regulating cell fate decisions (10–13). Notch signals are involved in the development and maintenance of normal tissues that are recapitulated in different forms of cancer (14,15). Notch can either promote or limit tumor growth, depending on the tumor type, through differentiation, cellular metabolism, cell cycle progression, angiogenesis and possibly self-renewal and immune function (16,17,19,20). The Notch signaling pathway is critical in cell fate decisions, tissue patterning and morphogenesis, and is hence regarded as a developmental pathway. However, problems with this pathway can contribute to cellular transformation and tumorigenesis.

The expression of Notch receptors and their downstream target genes is upregulated in primary human melanomas (15,16), and the expression of constitutively active Notch1 promotes melanoma progression (15,17). These oncogenic effects correlate with the activation of Wnt signaling in melanoma cells (15), which promotes the expression of adhesion molecules such as N-cadherin (17) through the transcription factor TCF/LEF (15). Notch has also been implicated in the pathogenesis of other solid tumors, such as medulloblastoma (18,19) and ovarian cancer (20), and the number of known neoplasms involving some alteration in Notch signaling is increasing. The aim of the present study was to assess whether vimentin and Notch gene and protein expression are altered in breast cancer progression. The importance of vimentin expression was analyzed by identifying cases of breast cancer with poor prognosis and comparing vimentin and Notch as biomarkers required for prognosis in breast cancer patients.

## Materials and methods

### Cell lines

MCF-10F cells were grown in DMEM/F-12 (1:1) medium supplemented with antibiotics [100 U/mI penicillin, 100 μg/ml streptomycin and 2.5 μg/ml amphotericin B (all from Life Technologies, Grand Island, NY, USA)] and 10 μg/m of 5% equine serum (Biofluids, Rockville, MD, USA), 0.5 μg/ml hydrocortisone (Sigma-Aldrich, St. Louis, MO, USA) and 0.02 μg/ml epidermal growth factor (Collaborative Research, Bedford, MA, USA) (21). An *in vitro* experimental breast cancer model (Alpha model) (22), developed by exposing the immortalized human breast epithelial MCF-10F cell line to low doses of high linear energy transfer α particle radiation (150 keV/μm) and subsequent growth in the presence or absence of 17β-estradiol, was used in this study. This model consisted of human breast epithelial cells in different stages of transformation: i) a control cell line (MCF-10F), ii) an Estrogen cell line [(MCF-l0F continually treated with estradiol at 10^−8^ M (Sigma-Aldrich)], iii) a malignant but non-tumorigenic cell line (Alpha3), iv) a malignant and tumorigenic cell line (Alpha5) and v) a Tumor2 cell line derived from cells originating from a tumor after injection of the Alpha5 cell line into nude mice. A total of 21 female CB17 SCID mice (Taconic, Germatown, NY, USA) and nude mice (Harlam Sprague Dawley, Indianapolis, IN, USA) (age, 1 year) were used in these studies. Each animal was injected subcutaneously at two different sites with 8×10^6^ cells in 0.2 ml saline in the fat pad of the right and left side of the abdominal mammary gland. The study was approved by the ethics committee of Columbia University Medical Center (New York, NY, USA)

### Pathological analysis

Formalin-fixed, paraffin-embedded, noninvasive and invasive ductal and lobular carcinomas were obtained from the archives of the Pathology Department of Dr Gustavo Fricke Hospital, Viña del Mar, Valparaíso, Chile. Patients had undergone surgery (total mastectomy with axillary lymph node dissection) between 1997 and 2001. The median patient age at surgery was 56 years (range, 25–92 years). The primary pathological diagnosis was confirmed by hematoxylin and eosin staining. All operative and pathological reports were reviewed to confirm disease stage. Sections of 2 μm were cut and mounted onto polylysine-coated slides, and stained for vimentin and Notch protein expression. The study was approved by the ethics committee of Dr. Gustavo Fricke Hospital of Viña del Mar (Valparaiso, Chile).

### Immunoperoxidase staining

Protein expression was evaluated as previously described (22–24). Exponentially growing cell lines were plated on a glass chamber slide (Nunc Inc., Naperville, IL, USA) at a density of 1×10^4^ cells/ml of medium and allowed to grow for 2–3 days until they reached 70% confluence (21). The cells were fixed with buffered paraformaldehyde at room temperature, incubated with 1% H_2_O_2_ in methanol to block endogenous peroxidase and washed twice with buffer solution. Cell cultures were subsequently covered with normal horse serum for 30 min at RT and incubated with anti-rabbit monoclonal antibody (vimentin: C-20, sc 7557 and Notch 4: C-19, sc 8644) (Santa Cruz Biotechnology, Inc., Santa Cruz, CA, USA) at a 1:500 dilution at 4ºC overnight, and then incubated for 45 min with diluted biotinylated secondary antibody solution (Vector Laboratories, Burlingame, CA, USA) and Vectastin Elite ABC Reagent (Vector Laboratories). The experiments were repeated three times in cells with identical passages *in vitro*. The number of immune-reactive cells (50 cells/field) was counted in several randomly selected microscopy fields (×400) per sample using an optical microscope (C×31; Olympus Corporation, Tokyo, Japan). Ten fields were counted for each cell line.

### Inmunofluorescent staining

Protein expression was evaluated by immunofluorescent staining and confocal microscopy as previously described (22,23). Cells were viewed on Zeis Axiovert 100 TV microscope (Carl Zeiss, Thornwood, NY, USA) using a 40× 11.3 NA objective lens equipped with a laser scanning confocal attachment (LSM 410, Carl Zeiss). A semi-quantitative estimation of the area and the intensity of the staining of the cells present in the culture dishes were performed based on the relative staining of the protein expressed by the controls and transformed cells.

### Fluorescent-labeled probe preparation for microarray analysis

The poly(A) mRNA from normal, radiation- and estrogen-treated breast cell lines was isolated using a QIA-direct-mRNA isolation kit (Qiagen, Inc., Valencia, CA, USA). Fluorescent-labeled cDNA was prepared from 1 μg of each of these poly(A) mRNA samples by using oligo dT-primed polymerization and a Superscript II reverse transcriptase kit (Life Technologies) in the presence of either Cy3- or Cy5-labeled dCTP, following the usual procedure (http://cmgm.stanford.edu/pbrown/protocols/). The appropriate Cy3- and Cy5-labeled probes were pooled and hybridized to microarray glass coverslips for 16 h at 65ºC and then washed with high stringency for analysis.

### Affymetrix HG-U133A Plus 2.0 GeneChip microarray gene expression analysis

The breast cancer model (Alpha model) containing i) MCF-10F, ii) Estrogen, (iii) Alpha3, iv) Alpha5 and v) Tumor2 cell lines was analyzed for gene expression using Affymetrix U133A oligonucleotide microarray (Affymetrix, Santa Clara, CA, USA), which contains 14,500 genes. Arrays were quantitatively analyzed for gene expression using the Affymetrix GeneChip Operating Software, with dual global scaling option in a Genes@Work software platform of discovery algorithm, Structural Pattern Localization Analysis by Sequential Histograms, and a false discovery rate of 0.05 (25,26).

## Results

### Phenotypic and molecular analysis of vimentin expression in breast cancer progression model

The established breast cancer model (22) has been shown to exhibit important phenotypic characteristics of breast carcinogenesis. The normal cell line, MCF-10F, did not exhibit any of the features that characterize malignant cells, such as anchorage-independent growth in soft agar, invasion and tumor growth in nude mice (22,24). The Alpha3 cell line formed colonies in soft agar and had invasive capabilities, but failed to form tumors in the immuno-suppressed mice. However, the Alpha5 cell line induced mammary gland tumors in the animals and metastasis in the liver, lung and kidneys after injection. This cell line gave rise to the Tumor2 cell line after removal of the mammary tumor, digestion in *in vitro* conditions and culture for many passages.

**Figure 1:**
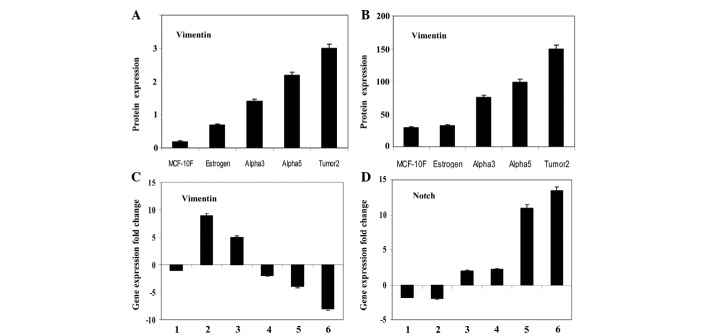
Bars represent the average and standard error of vimentin protein expression by (A) peroxidase and (B) immunofluorescent techniques of the MCF-10F, Estrogen, Alpha3, Alpha5 and Tumor2 cell lines. The primary antibodies used were mouse monoclonal antibodies (Santa Cruz Biotechnology, Inc., Santa Cruz, CA, USA). Fold change of (C) *vimentin* and (D) *Notch* gene expression. Gene expression from scatter plots of the following pairwise comparative studies of cell lines: MCF-10F/E (1), MCF-10F/Alpha3 (2), E/Alpha5 (3), Alpha3/Alpha5 (4), Alpha 3/Tumor2 (5) and Alpha5/Tumor2 (6).

The analysis of immunoperoxidase (Fig. 1A) and immunofluorescence (Fig. 1B) data obtained in relation to the relative vimentin expression in MCF-10F, Estrogen, Alpha3, Alpha5 and Tumor2 cell lines indicated that such expression was significantly greater (P<0.05) in the Tumor2, Alpha3 and Alpha5 cell lines, when compared with the MCF-10F and Estrogen cell lines. Genes that were identified to be differentially expressed between cell lines of this model were also studied. Histogram plots of the differential expression of vimentin and Notch genes in these cell lines were detected by gene chip array. Results of pairwise comparisons of cell lines examined for vimentin protein expression were analyzed with the following pairs of cell lines: MCF-10F/Estrogen, MCF-10F/Alpha3, Estrogen/Alpha5, Alpha3/Alpha5, Alpha5/Tumor2 and Alpha 3/Tumor2 (Fig. 1C). Results of the pairwise comparisons did not reveal any alteration in vimentin gene expression between the MCF-10F and Estrogen cell lines, while there was an almost nine- and five-fold alteration in the MCF-10F/Alpha3 and Estrogen/Alpha5 combinations, respectively. There were six- and four- fold changes in gene expression between the Alpha5 and Tumor2 cell lines, and Alpha3 and Tumor2 cell lines, respectively.

Results of pairwise comparisons of cell lines examined for *Notch* gene expression are shown in Fig. 1D. Results of the same pairs of cell lines were analyzed, revealing no alteration in Notch gene expression between the MCF-10F and Estrogen cell lines, Estrogen and Alpha5 cell lines, and Alpha3 and Alpha5 cell lines. By contrast, there was an almost ten- and fourteen-fold alteration in the Alpha5/Tumor2 and Alpha3/Tumor2 combinations, respectively, with higher expression in Alpha3 and Alpha5 than in Tumor2.

### Vimentin protein expression in breast cancer model and breast biopsy specimens

**Figure 2:**
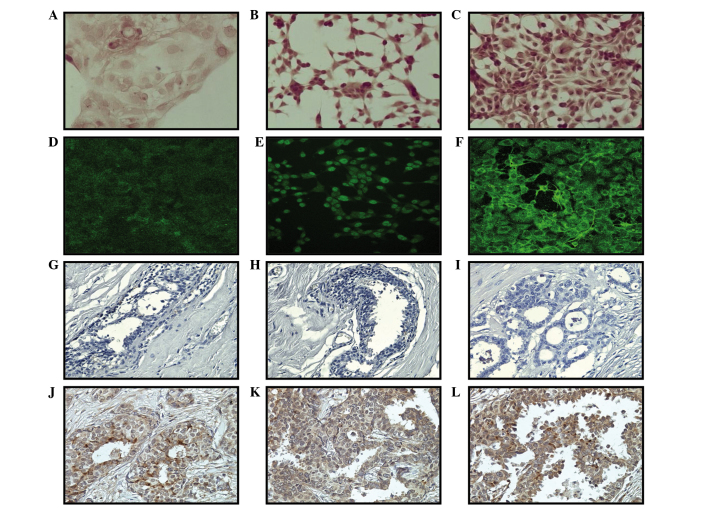
Representative images of vimentin protein expression stained by (A–C) immunoperoxidase and (D–F) immunofluorescent techniques in a breast cancer cell model. Biopsy specimens containing (G–I) ducts and lobules, and (J–L) invasive carcinoma determined by inmunoperoxidase techniques (magnification, ×400).

Representative images of vimentin protein expression, in which greater expression was observed in the Alpha5 and Tumor2 cell lines compared with that in the control MCF-10F cell line, can be observed in immunoperoxidase (Fig. 2A–C) and immunofluorescence (Fig. 2D–F) studies. Biopsy specimens were also analyzed for vimentin protein expression to analyze progression in breast cancer. Fig. 2G–I shows representative tissues of vimentin protein expression in ducts found in sections of biopsies from breast cancer patients, as determined by immunoperoxidase staining. This expression was negative in noninvasive ductal carcinoma and breast epithelial lesions surrounding the primary tumors, ductal and lobular hyperplasia, and microcytes. By contrast, this expression was positive in breast specimens with invasive characteristics, as shown in Fig. 2J–L. Positive staining for vimentin was found in 21% of cases.

### Notch protein expression in breast cancer model and breast biopsy specimens

**Figure 3:**
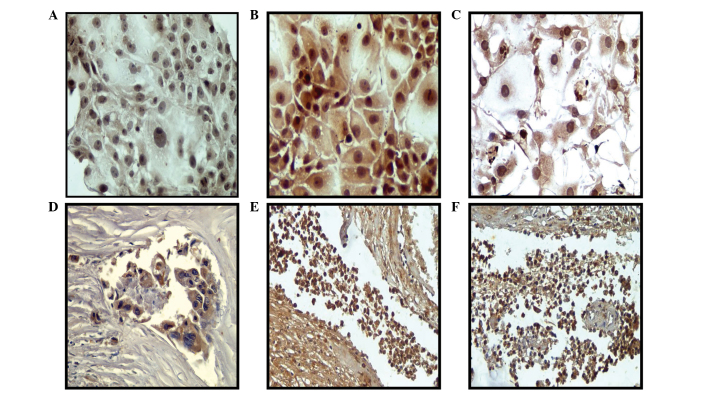
Notch protein expression of (A) MCF-10F; (B) Alpha5; (C) Tumor2 cell lines. Biopsy specimens containing (D) ductal carcinoma and (E and F) invasive ductal carcinoma determined by immunoperoxidase technique (magnification, ×400).

In the present study, non-malignant and malignant cell lines from the model were used to analyze Notch protein expression. Fig. 3A–C shows higher Notch protein expression in the Alpha5 and Tumor2 cell lines compared with that in the control MCF-10F cell line, as determined by immunoperoxidase staining. Samples from biopsy specimens showed negative Notch protein expression in noninvasive ductal carcinomas. However, positive cell expression was observed in those tissues with cells from invasive ductal carcinomas (Fig. 3D–F), particularly in invasive isolated tumor cells. Positive staining for Notch was found in 25% of cases.

## Discussion

The main purpose of the present study was to assess the prognostic value of the markers vimentin and Notch. Identification of factors involved in cell proliferation and transformation has been facilitated by studies using various human epithelial cell lines. The analysis of immunoperoxidase and immunofluorescence data obtained in relation to the relative *vimentin* expression indicated that such expression was significantly greater in Tumor2 and Alpha5 when compared with MCF-10F, Estrogen and Alpha3 cell lines.

Results of pairwise comparisons of *vimentin* gene expression in the different cell lines indicated that there was no alteration in vimentin gene expression between the MCF-10F and Estrogen cell lines, while there was an almost nine- and five-fold alteration in the MCF-10F/Alpha3 and Estrogen/Alpha5 combinations, respectively. There were six- and four-fold changes in gene expression between Alpha5 and Tumor2, and Alpha3 and Tumor2, respectively. Results of the same pairs of cell lines analyzed for *Notch* gene expression indicated that there was no alteration between the MCF-10F and Estrogen, Estrogen and Alpha5, and Alpha3 and Alpha5 cell lines. By contrast, there was an almost ten- and fourteen- fold alteration in the Alpha5/Tumor2 and Alpha3/Tumor2 combinations, respectively, with higher expression in Alpha3 and Alpha5 than in Tumor2 cells. Vimentin protein expression in ducts in sections of biopsies from breast cancer patients was found to be negative for noninvasive ductal carcinoma, but positive for ductal carcinoma with invasive characteristics. Vimentin-reactive cells in benign and malignant breast tissue have been described in many studies (26–29). These studies reported that vimentin expression appeared to be associated with poor prognosis in node-negative ductal breast carcinomas, and that vimentin was preferentially expressed in human breast carcinomas with low levels of estrogen receptors. Gene expression patterns of breast carcinomas distinguished tumor subclasses with clinical implications (30). A possible association was found between the clinically aggressive behavior of tumors (28,29) and estrogen receptor negativity (31,32), high Ki-67 levels (32) and poor differentiation of tumors with high-grade and positive vimentin protein expression. Domagala *et al* (29) reported that vimentin was preferentially expressed in high-grade ductal and medullary, but not in lobular, breast carcinomas. Other data showed that more invasive breast cancer lines expressed vimentin, indicating its usefulness in identifying cases with poorer prognosis (28,29).

Vimentin is known to be selectively expressed in aggressive breast cancer cell lines (9). Elevated *vimentin* expression levels correlate well with upregulated migration and invasion of cancer cells (9,26). Sommers *et al* (27) showed that transfection of noninvasive human breast cancer cell lines, such as MCF7, with the vimentin gene led to accelerated invasiveness. The authors also reported vimentin rather than keratin expression in certain hormone-independent breast cancer cell lines, and in oncogene-transformed mammary epithelial cells. The possible association of vimentin with the clinically aggressive behavior of tumors described by others (7,28–32) may be explained by the correlation of vimentin expression with a lack of steroid receptors and poor differentiation of cancer. Gilles *et al* (31) also found vimentin expression in cervical carcinomas was associated with invasive and migratory potential.

Thus, we can suggest an improved indicator of breast cancer progression by adding vimentin to the diagnostic panel when overall survival is a primary end-point. In the present study, positive staining for vimentin was found in 21% of cases, which is in line with previous findings (32). Therefore, vimentin expression appears to predict survival in ductal breast carcinoma.

Notch protein expression was also higher in the Alpha5 and Tumor2 cell lines in comparison with that in the control MCF-10F cell line. When samples from biopsy specimens were analyzed for Notch protein expression, negative cells were found in noninvasive ductal carcinomas while positive cells were found in invasive ductal carcinomas. It has been reported that the Notch pathway is required for the establishment of embryonic hematopoietic stem cells (33), and it has been implicated in the maintenance of several types of normal cell populations (34–36). The effects of Notch on cells include increased survival or death, proliferation or growth arrest and commitment to, or blockage of, differentiation. These different outcomes are mediated through a novel signaling pathway in which Notch receptors on the cell surface give rise to a nuclear transcriptional activation complex. Studies on Notch are related to the understanding of how this pathway yields several outcomes. It has been proposed that Notch may serve as an oncogene or tumor suppressor, a repressor or inducer of terminal differentiation, or a cancer stem cell factor. Studies on the multifaceted role of Notch in cancer indicate a possible therapeutic implication. Notch signaling is frequently deregulated in breast cancer, and hyperactivation of Notch contributes to the tumor process. Notch has been shown to be involved in the controlled proliferation and migration of vascular endothelial cells, as well as in the integration of Notch and Wnt signaling, as observed in hematopoietic stem cell maintenance (34). Guentchev and McKay (35) observed that Notch controlled the proliferation and differentiation of stem cells in a dose-dependent manner. It has also been suggested that Notch acts as a transducer molecule for developmental processes. Stylianou *et al* (36) observed aberrant activation of Notch signaling in human breast cancer.

Estrogens are known to regulate the proliferation of breast cancer cells and to alter their phenotypic properties; the gene networks and pathways through which estrogenic hormones regulate these events have also been considered (37). We used global gene expression profiling by Affymetrix GeneChip microarray analysis to identify genes altered by the presence of estradiol in an MCF-10F human breast cancer model. Of the >14,000 genes analyzed, over 300 showed a pattern of either up- or downregulation. We observed a general upregulation of positive proliferation regulators, including multiple growth factors, genes involved in cell cycle progression and regulatory factor-receptor loops, and a downregulation of transcriptional repressors and anti-proliferative and pro-apoptotic genes, including *BCL2* and *TGF-β* family growth inhibitory factors. The present study highlights the diverse gene networks and metabolic and cell regulatory pathways through which this hormone operates to achieve its widespread effects on breast cancer cells.

It can be concluded that *vimentin* and *Notch* gene and protein expression are altered in breast cancer progression, thereby helping to identify cases of breast cancer with poor prognosis and complementing those biomarkers required for assessing the prognosis of breast cancer patients.
